# Ratio of Extracellular to Intracellular Water Is Associated with Permanent Catheter Patency Survival in Patients Receiving Maintenance Hemodialysis

**DOI:** 10.3390/diagnostics13152545

**Published:** 2023-07-31

**Authors:** Moo-Jun Kim, Jae-Wan Jeon, Hae-Ri Kim, Hyerim Park, Suyeon Han, Yunkyeong Hwang, Heewon Park, Kyungho Park, Eu-Jin Lee, Young-Rok Ham, Ki-Ryang Na, Kang-Wook Lee, Dae-Eun Choi

**Affiliations:** 1 Department of Nephrology, Chungnam National University Sejong Hospital, Sejong 30099, Republic of Korea; kimmoojun@cnuh.co.kr (M.-J.K.); jeonjwan@cnuh.co.kr (J.-W.J.); yo0118@cnuh.co.kr (H.-R.K.); 2 Department of Medical Science, Chungnam National University, Daejeon 35015, Republic of Korea; hye05240@gmail.com; 3 Department of Nephrology, Chungnam National University Hospital, Daejeon 35015, Republic of Korea; garlic1208@cnuh.co.kr (S.H.); hyk5210@gmail.com (Y.H.); heewon910125@gmail.com (H.P.); ds3ixd@gmail.com (K.P.); eujinlee@cnuh.co.kr (E.-J.L.); youngrok01@cnuh.co.kr (Y.-R.H.); kwlee@cnu.ac.kr (K.-W.L.)

**Keywords:** body composition monitoring, extracellular water, intracellular water, hemodialysis, permanent catheter, catheter patency, infection, malfunction

## Abstract

Patients undergoing dialysis through a permanent catheter often experience infection or malfunction. However, few studies have clarified the predictors of permanent catheter patency survival in patients undergoing hemodialysis. We assessed the relationship between the parameters of body composition monitoring (BCM), determined before the initiation of dialysis, and the patency survival of the permanent catheters inserted in 179 patients who commenced hemodialysis between 14 January 2020 and 31 August 2021. The relationships between permanent catheter patency at 6 weeks and BCM parameters, laboratory tests, age, sex, comorbidities, and medications at baseline were studied using Kaplan–Meier survival curves. Permanent catheter patency was observed to be superior at high extracellular-to-intracellular (ECW/ICW) ratio (*p* < 0.005). After adjustment for covariates, the ECW/ICW ratio remained an independent factor associated with permanent catheter patency survival. When patients with non-patent catheters were subdivided into infection and malfunction groups, and the associations of BCM parameters were evaluated in those groups, the ECW/ICW ratio was not significantly associated with permanent catheter patency survival in the infection group (*p* = 0.327); instead, a significant association was found for the lean tissue index (*p* < 0.001). In the malfunction group, the ECW/ICW ratio remained significantly associated with permanent catheter patency survival (*p* < 0.001).

## 1. Introduction

The parameters of body composition monitoring (BCM) include information such as volume status, lean tissue mass, fat mass (FAT), and dry weight [1]. The BCM parameters relating to fluid management for patients receiving hemodialysis (HD) have been extensively examined in both observational and experimental studies [2,3]. Although the best vascular access for HD treatment is an arteriovenous fistula, patients commencing dialysis often require the insertion of a permanent catheter into the internal jugular vein when a vascular access has not yet been prepared [4,5]. However, the use of a permanent catheter during HD carries a higher complication rate than do other options for vascular access such as arteriovenous fistulae and synthetic vascular grafts [6]. During the use of a permanent catheter for dialysis initiation and maintenance, patients often experience malfunction and infection of the catheter [7,8]. Several previous studies examined the extracellular-to-total body water (ECW/TBW) and extracellular-to-intracellular water (ECW/ICW) ratios in patients receiving maintenance HD [9,10,11,12,13,14]. However, fewer studies have investigated the association of permanent catheter patency survival with those parameters. We therefore designed a study to investigate the association between the BCM parameters measured at the initiation of HD and the occurrence of catheter malfunction and infection. Our aim was to gain information about safety and complication prevention in patients receiving HD through a permanent catheter.

## 2. Material and Method

### 2.1. Study Design and Participants

**Figure 1:**
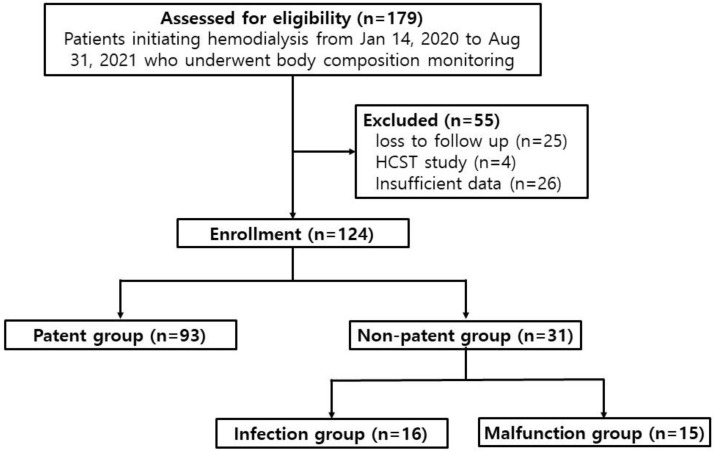
Study design. HSCT: hematopoietic stem cell transplantation.

We retrospectively reviewed the records of 179 patients who had commenced dialysis with a permanent catheter between 14 January 2020 and 31 August 2021, at Chungnam National University Hospital (Figure 1). Patients were eligible for enrollment if they were between the ages of 19 and 95 years, and if they had undergone blood sampling and BCM before the start of renal replacement therapy. Patients with end-stage renal disease who started hemodialysis for the first time due to unresolved uremic symptoms including diuretic resistant edema or gastrointestinal symptom were included. This study did not include patients whose urine output was more than 500 mL/day even after 7 days, which meant dialysis was performed 5 times for 7 days. It was not based on the first day’s urine volume, but patients with persistent oliguria with urine output < 500 mL/day after 7 days of dialysis were included in the study. Since only patients newly starting dialysis were targeted, these patients did not have a previous history of catheter insertion. Of the 179 patients reviewed, 55 were excluded because they either were lost to follow-up (n = 25), had been studied for other purposes (hematopoietic stem cell transplantation, n = 4), or lacked sufficient data for analysis (n = 26). When recruiting participants, some patients died during the follow-up period, and most of these patients died not due to abnormal catheter patency, but due to aggravation of other underlying diseases. This could cause a statistical error when evaluating catheter patency, so deceased patients were preemptively excluded from the patient recruitment stage. Of the 124 eligible patients, 93 who had used their permanent catheter continuously for six weeks without abnormalities were designated the patent group. The remaining 31 patients who had experienced problems using the permanent catheter during the first six weeks were designated the non-patent group. In the latter group, patients with a confirmed catheter infection were designated the infection group (n = 16), and patients whose HD could not be regularly performed using the catheter were designated the malfunction group (n = 15).

Information about the study cohort collected from medical records included age, sex, type of dialysis catheter, duration of renal replacement therapy through the dialysis catheter (days), underlying disease (hypertension, diabetes mellitus, cardiovascular disease, cerebral infarction, liver cirrhosis), cause of end-stage renal disease, medication history (aspirin, clopidogrel, warfarin, cilostazol, non-vitamin K antagonist oral anticoagulants, statins), operation date, catheter follow-up management, reason for catheter removal, catheter removal date, catheter usage period, BCM parameters—ECW, ICW, TBW, ECW/ICW ratio, lean tissue index (LTI), fat tissue index (FTI), lean tissue mass, adipose tissue mass, FAT, dry weight—laboratory tests (hemoglobin, platelets, C-reactive protein, ferritin, parathyroid hormone, total protein, serum albumin, blood urea nitrogen, creatinine, total calcium, phosphate, sodium, potassium), pre-HD weight, post-HD weight, dialysis duration, and ultrafiltration. A BCM device (Fresenius Medical Care, Bad Homburg, Germany) was used to evaluate body composition parameters before dialysis on the day of dialysis.

Ultrafiltration and blood flow were set using the BCM parameters performed before dialysis as indicators; it was difficult to unify them uniformly because they were personalized according to the patient’s condition. In addition, since only patients newly starting dialysis were targeted, these patients did not have a previous history of catheter insertion.

This study was conducted in accordance with the 1964 Declaration of Helsinki and its later amendments. Medical records were retrieved anonymously in accordance with data protection rules after approval had been obtained from the hospital’s ethics committee. The institutional review board of Chungnam National University Hospital approved the study (no. 2022-12-074).

### 2.2. Definitions

#### 2.2.1. Infection of Permanent Catheter

An infection of the permanent catheter was accepted as follows:

- when redness, tenderness, warmth, or pus was observed at the catheter exit site, and infection was simultaneously suspected from laboratory tests.
- when no infection was evident before catheter insertion, but symptoms of infection such as fever began within five days after insertion, infection was confirmed on blood culture, and no prominent infection in other organs was evident.

#### 2.2.2. Malfunction of Permanent Catheter

A malfunction of the permanent catheter was accepted as follows:

- when an extracorporeal blood flow sufficient to perform HD (<300 mL/min) could not be attained or maintained.
- when catheter dysfunction unrelated to catheter tip malposition or mechanical kinking of the catheter occurred.

### 2.3. Statistical Analysis

The IBM SPSS Statistics software application (version 26: IBM, Armonk, NY, USA) was used for the analyses, and *p* < 0.05 was considered to be statistically significant.

An independent *t*-test was used to determine whether each BCM parameter had a significant association with permanent catheter patency survival in each group. A subsequent analysis of the area under the receiver operating characteristic curve (AUC-ROC) was used to determine the best cut-off values for the parameters that were significant in the independent *t*-tests. The cut-off values thus obtained in the non-patent group were used to define subgroups below and above the cut-off values. Continuous use of the permanent catheter during the initial six-week usage period was then plotted using Kaplan–Meier curves to assess the differences between the groups.

Multivariate Cox regression adjusted for age and sex was performed to verify whether the cut-off values of significant parameters in the non-patent group were also significant in the infection and malfunction subgroups. Adjusted hazard ratios and 95% confidence intervals were calculated.

## 3. Results

Table 1 summarizes participant characteristics at baseline. The study population included 68 men (54.8%; median age: 72 years) and 56 women (45.2%; median age: 72 years), with the patent group consisting of 93 patients, and the non-patent group consisting of 31 patients. The median age in the patent and non-patent groups was 69 years and 75 years respectively. Both groups had high rates of diabetes mellitus and hypertension as comorbidities.

The independent sample *t*-tests performed to determine whether the patent and non-patent groups differed significantly in BCM parameters and the predialysis blood tests found significant differences in the ICW, TBW, ECW/ICW ratio, LTI, FTI, lean tissue mass, FAT, and BCM (*p* < 0.05), as well as in predialysis serum C-reactive protein, albumin, and creatinine (*p* < 0.05). Supplementary Table S1a,b presents the baseline characteristics of the infection and malfunction subgroups within the non-patent group.

**Figure 2:**
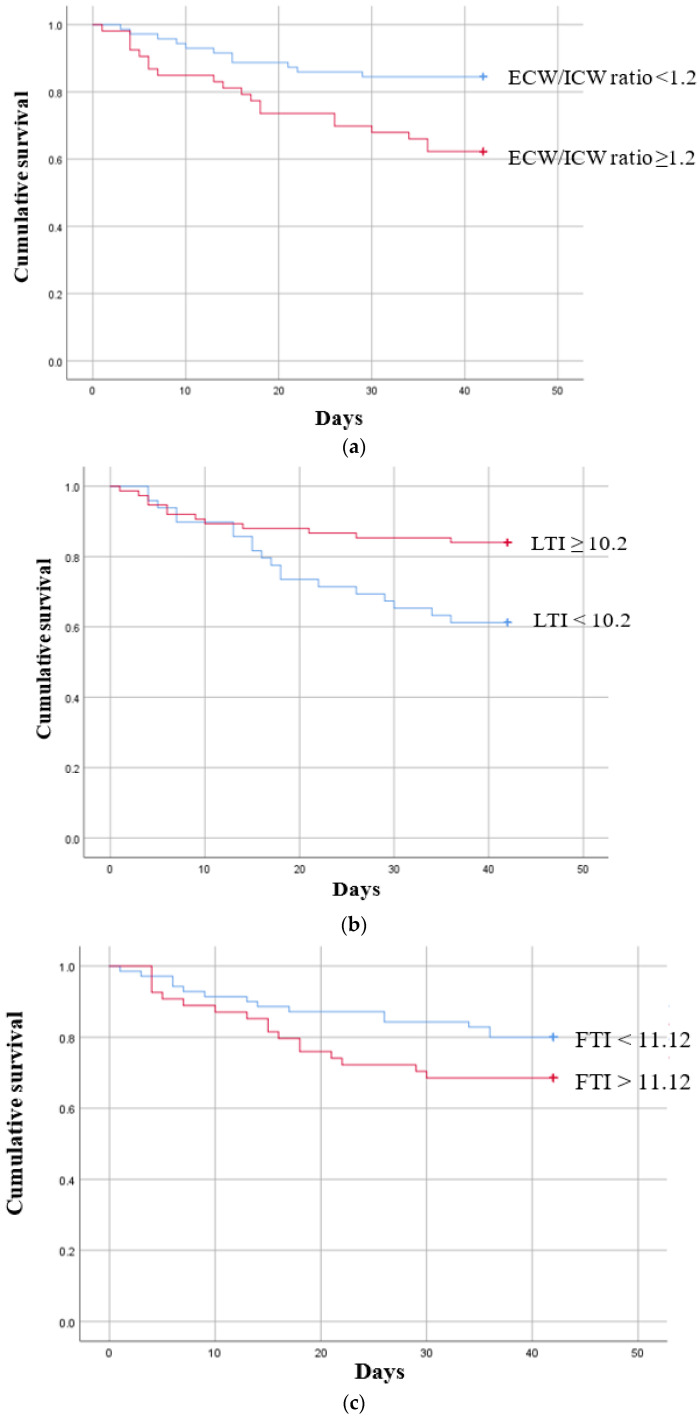
Kaplan–Meier survival curves showing the six-week permanent catheter survival for patients in the patent and non-patent groups using the cut-off values obtained for significant body composition monitoring parameters by receiver operating characteristic curve analysis. (**a**) ECW/ICW ratio (n = 124) log-rank χ^2^ = 7.797 *p* = 0.005, (**b**) LTI (n = 124) log-rank χ^2^ = 7.425 *p* = 0.006 (**c**) FTI (n = 124) log-rank χ^2^ = 2.233 *p* = 0.135. ECW: extracellular water; ICW: intracellular water; LTI: lean tissue index; FTI: fat tissue index.

ROC curves were then calculated to confirm the types of associations that the ECW/ICW ratio, LTI, and FTI had between the groups (Supplementary Figure S1a–c). Based on the cut-off values obtained from the ROC curves, Kaplan–Meier survival curves were plotted for six-week permanent catheter patency survival in the participants below and above the cut-off value (Figure 2a–c). In the case of the ECW/ICW ratio, the AUC was determined to be 0.701, and after the cut-off value of 1.20 obtained from the ROC-AUC analysis was used to allocate participants to a “below” or “above” group (Supplementary Figure S1a), the Kaplan–Meier curves were significantly different (log-rank *p* < 0.005, Figure 2a), demonstrating that the patency of the permanent catheter was reduced in the group with a high ECW/ICW ratio. The AUC for LTI was 0.723, and when the cut-off value of 11.12 obtained from the ROC-AUC analysis was used to create Kaplan–Meier curves for the “below” and “above” groups (Supplementary Figure S1b), the difference was significant (log-rank *p* = 0.006, Figure 2b), showing that permanent catheter patency survival was reduced in the group with the low LTI. The AUC for FTI was 0.625, and when the cut-off value of 11.12 obtained from the ROC-AUC analysis was used to create Kaplan–Meier curves for the “below” and “above” groups (Supplementary Figure S1c), the difference was nonsignificant (log-rank *p* = 0.135, Figure 2c).

A univariate Cox proportional hazards analysis showed that age, ICW, ECW/ICW ratio, LTI, FTI, lean tissue mass, FAT, and body cell mass were all significantly associated with the patency of the permanent catheter (Supplementary Table S2). On multivariate Cox regression analyses, when other variables (age, sex, LTI, FTI) were added in stages, the hazard ratio for the association between the ECW/ICW ratio and permanent catheter patency remained highly significant (Table 2).

**Figure 3:**
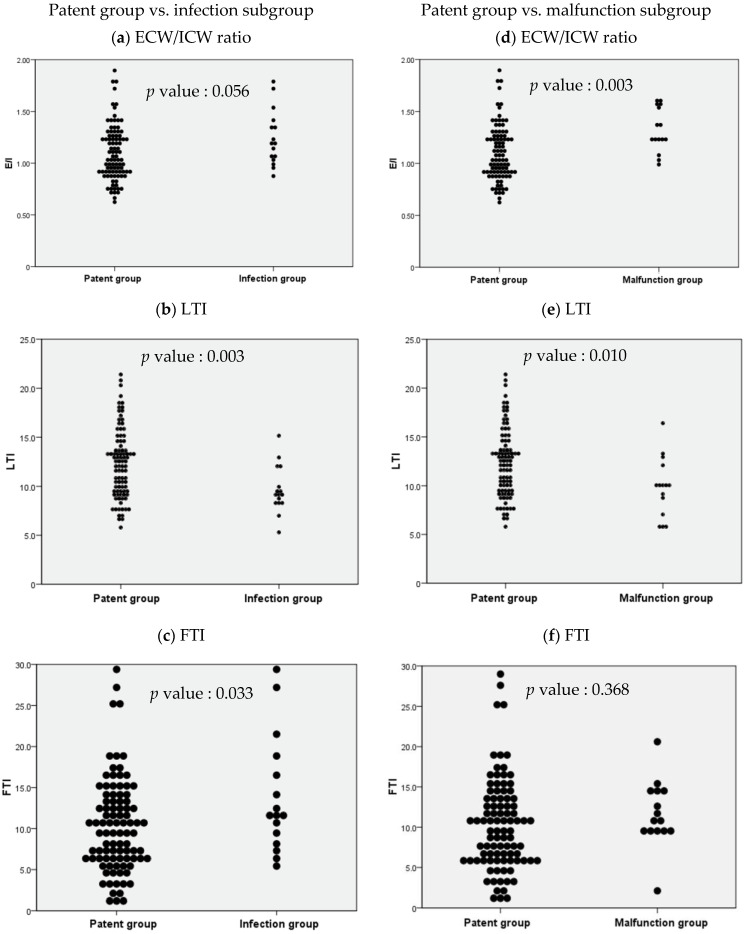
Dot plots based on the independent sample *t*-test results for body composition monitoring parameters between selected study groups. ECW: extracellular water; ICW: intracellular water; LTI: lean tissue index; FTI: fat tissue index.

When independent sample *t*-tests were performed to compare BCM parameters in the patent group and the infection subgroup, ICW, LTI, FTI, FAT, and body cell mass were observed to be significantly different, and when independent sample *t*-tests were performed to compare BCM parameters in the patent group and the malfunction subgroup, ICW, ECW/ICW ratio, LTI, and body cell mass were observed to be significant (Supplementary Table S2a,b). Distribution tables for the significant parameters ECW/ICW ratio, LTI, and FTI were then expressed as dot plots to examine the degree of distribution (Figure 3).

The independent sample *t*-test comparing the ECW/ICW ratio in the patent group and the infection subgroup resulted in a *p* value of 0.056, which was nonsignificant, but the comparison between the patent group and the malfunction subgroup resulted in a *p* value of 0.003, which was significant (Figure 3a,d). Similar comparisons of LTI in the patent group and the non-patent subgroups resulted in *p* values of 0.003 and 0.010 respectively (both statistically significant, but stronger in the infection subgroup, Figure 3b,e). Similar comparisons of FTI resulted in *p* values of 0.033 and 0.368 respectively (significant in the infection subgroup, but nonsignificant in the malfunction subgroup, and weak compared with the other parameters; Figure 3c,f).

**Figure 4:**
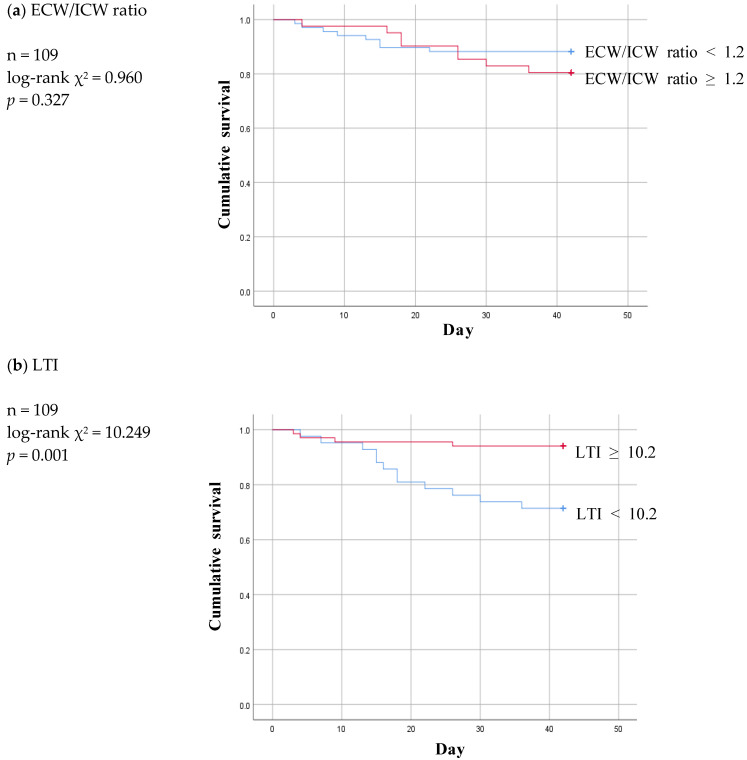

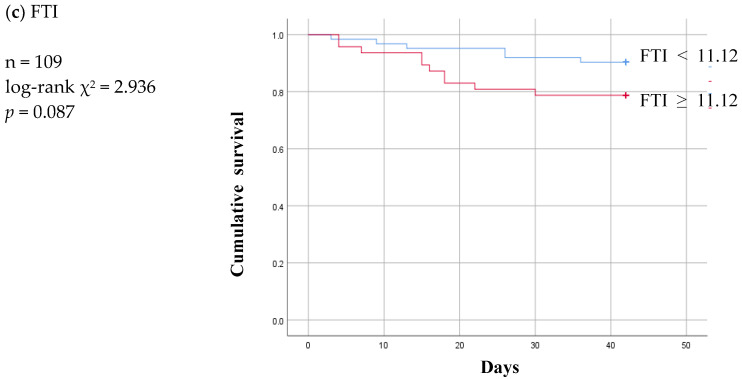
Six-week Kaplan-Meier survival curve for patients of patent and infection subgroup using the cut-off values for various body composition monitoring parameters obtained by ROC analysis in non-patent group. ECW: extracellular water; ICW: intracellular water; LTI: lean tissue index; FTI: fat tissue index.

**Figure 5:**
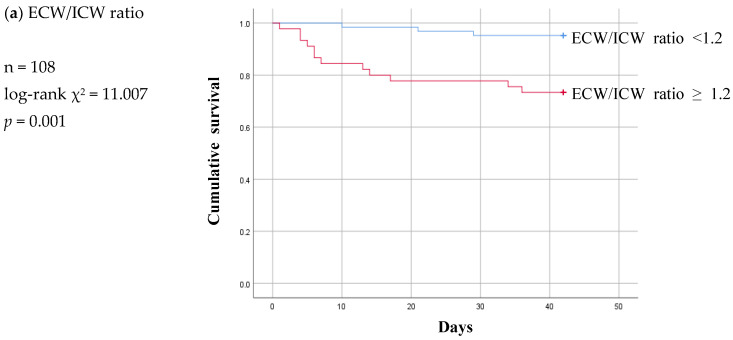

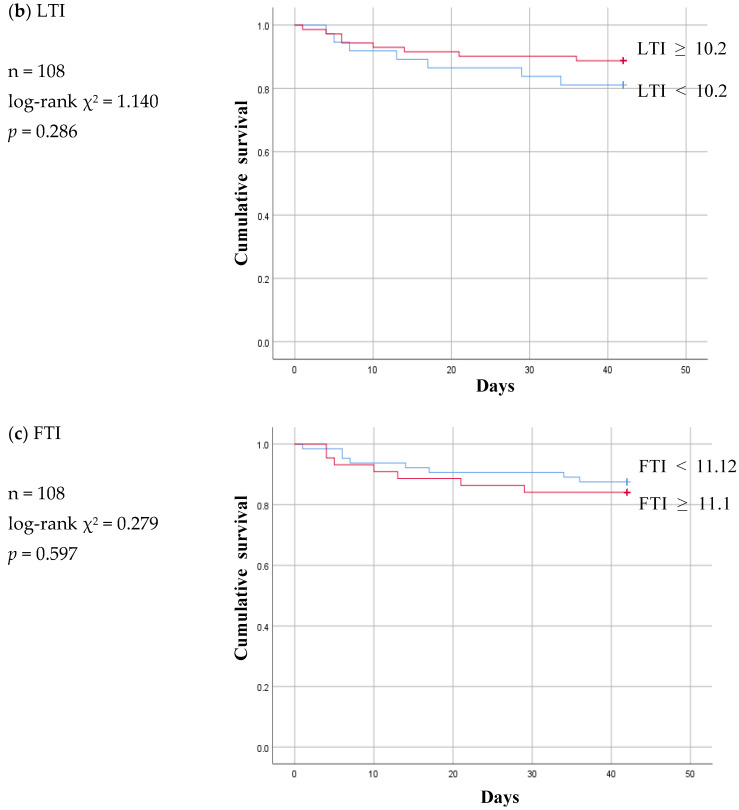
Six-week Kaplan-Meier survival curve for patients of patent and malfunction subgroup using the cut-off values for various body composition monitoring parameters obtained by ROC analysis in non-patent group. ECW: extracellular water; ICW: intracellular water; LTI: lean tissue index; FTI: fat tissue index.

Based on the cut-off values for the foregoing three parameters, six-week Kaplan–Meier survival curves were then plotted for the “below” and “above” groups in the two non-patent subgroups and compared with those for the non-patent group (Figure 4 and Figure 5).

Of the three parameters, LTI ≥ 10.2 remained significant at a *p* value of 0.001 and ECW/ICW ratio < 1.2 lost statistical significance at a *p* value of 0.327 in the infection subgroup (Figure 4a,b). At a *p* value of 0.087, FTI was nonsignificant in the infection subgroup just as it had been in the non-patent group (Figure 4c).

The Kaplan–Meier curve pattern was different in the malfunction subgroup compared to the infection subgroup. LTI and FTI lost their significance, and the ECW/ICW ratio became significant at a log-rank *p* value of 0.001 (Figure 5).

A Kaplan–Meier survival curve was also plotted using the controlling nutritional status score (cut-off value: 3 points) to analyze permanent catheter patency survival based on nutrition status. The log-rank *p* values obtained for the non-patent group, the infection subgroup, and the malfunction subgroup were 0.380, 0.486, and 0.135 respectively. No significant correlation between nutrition status and the patency survival of the permanent catheter was observed (Supplementary Figure S2).

## 4. Discussion

Problems with permanent catheter patency are often caused by infection and malfunction [15,16,17]. Protein–energy expenditure, inflammation, and fluid overload are well-known independent predictors of death in dialysis patients [18,19]. Wizemann et al. found that baseline overhydration in HD patients with an OH level above 2.5 L (OH:ECW ratio > 15%) is also associated with a significantly increased risk of mortality [20]. Furthermore, Zhou et al. found that the ECW/ICW ratio is associated with increased sarcopenia risk independent of body mass index, pre-albumin, C-reactive protein, and other potential confounders in patients on maintenance HD [11]. However, few studies have attempted to clarify the predictors of permanent catheter patency survival in patients undergoing HD. Results from the present study demonstrate that the ECW/ICW ratio is a marker for the patency survival of a permanent catheter. The parameters ECW/ICW ratio < 1.2 and LTI ≥ 10.2 could be useful in predicting whether infection or malfunction might affect permanent catheter patency before it becomes a problem. Ohashi et al. and Dekker et al. found that the balance between the ICW and ECW volumes is significantly associated with age. The ECW/ICW ratio is thus understood to be primarily driven by decreasing cell volume—and particularly its steepening trend after the age of 70 years. The balance between ICW and ECW might therefore change when the hydration component gradually decreases with muscle attenuation [21,22]. In the present study, the multivariate Cox proportional hazard analyses revealed that not only the ECW/ICW ratio, but also age was statistically significant, which again demonstrates that the ECW/ICW ratio is correlated with age.

Application of the ECW/ICW ratio has attracted much attention in the past in diseases other than chronic renal disease. An increased ECW/ICW ratio is reported to be associated with the level of albuminuria in patients with type 2 diabetes mellitus [14] and with greater functional disability in patients with knee osteoarthritis [13]. Furthermore, the ECW/ICW ratio is a major risk indicator for all-cause mortality and cardiovascular disease in patients receiving HD or peritoneal dialysis [23,24]. In the present work, we demonstrated that the ECW/ICW ratio is powerfully associated with the patency survival of permanent catheters for HD. A high ECW/ICW ratio signals a decrease in ICW and a relative increase in ECW. There is speculation that fluid moves to the extravascular space as a result, narrowing the intravascular space and causing frequent contact between the permanent catheter and the vessel wall. The trauma caused by this contact, with its increased inflammation, activation of leukocytes, and release of myeloperoxidase, activates the coagulation cascade, which is thought to trigger catheter malfunction [25,26]. In our infection group, permanent catheter patency survival declined in the group having an LTI below our estimated cut-off value. Lower LTI presents as less protein storage and a low energy reserve, both of which are necessary for metabolic processes [27], possibly making patients more vulnerable to infection. When the permanent catheter becomes infected, inflammatory cells colonize the catheter and might play a role in venous thrombosis [28]. Initiation of the coagulation and inflammatory response cascade activates leukocytes, which release myeloperoxidase to form platelet aggregates. A soft clot could then form on or inside the catheter tip, causing dysfunction [26].

We should acknowledge that our study has several limitations. First, it is a single-center retrospective review that relies on available patient information; it may contain potential incomplete or missing information, selection bias, and inability to establish causal relationships. Generalization of our results to another population undergoing evaluation might therefore not be valid. Second, we did not evaluate racial or ethnic differences between the participants because only South Korean individuals were included in the study. It is hoped that if additional studies are conducted in other countries or other medical centers and BCM parameters show significant results on catheter patency, it will be a more powerful indicator. Third, despite adjusting for some covariates, it is possible that unmeasured or unaccounted factors could influence the outcomes and confound the observed associations. Fourth, we could not evaluate the permanent catheter patency survival based on the association between sarcopenia and the ECW/ICW ratio. Because of the retrospective nature of the study and the fact that the participants were chiefly patients who required a permanent catheter because of their need for immediate dialysis, a work-up for sarcopenia was, regrettably, impossible. If additional studies into differences in permanent catheter patency survival according to BCM parameters are conducted in the future, determining any correlations with sarcopenia and racial differences will be of great help in the management of patients undergoing maintenance HD.

## 5. Conclusions

BCM parameters obtained before the start of dialysis are expected to play an important role in predicting the prognosis for permanent catheter patency survival. Specifically, identifying and managing the patient population with a high ECW/ICW ratio and a low LTI is expected to be of great help in the future treatment of patients receiving maintenance HD. Additional long-term prospective studies in other cohorts are needed to confirm the usefulness of the ECW/ICW ratio and LTI parameter proposed in the present study as important predictors of permanent catheter patency survival. It is hoped that catheter patency studies according to the BCM parameter will be conducted when dialysis is started in medical centers in other countries where various races can be treated. This is expected to be of great help in the management of patients who maintain dialysis treatment through a permanent catheter.

## Figures and Tables

**Table 1:** Baseline characteristics of the study participants, by catheter patency.

Characteristic	Overall (N = 124)	Catheter Group		*p* Value
		Patent (N = 93)	Non-Patent (N = 31)	
Age (median [range] years)	72.0 [26–93]	69.0 [26–93]	75.0 [50–92]	0.003
Sex [n (%)]				
Men	68 (54.8)	54 (58.1)	14 (45.2)	0.214
Women	56 (45.2)	39 (41.9)	17 (54.8)	
Comorbidities [n (%)]				
Diabetes mellitus	89 (71.8)	69 (74.2)	20 (64.5)	0.304
Hypertension	96 (77.4)	68 (73.1)	28 (90.3)	0.048
Heart failure	41 (33.1)	31 (33.3)	10 (32.3)	0.913
Ischemic heart disease	34 (27.4)	28 (30.1)	6 (19.4)	0.249
Atrial fibrillation	24 (19.4)	15 (16.1)	9 (29.0)	0.117
Cerebral infarction	19 (15.3)	10 (10.8)	9 (29.0)	0.014
Liver cirrhosis	6 (4.8)	1 (1.1)	5 (16.1)	0.001
Medication [n (%)]				
Aspirin	29 (23.4)	20 (21.5)	9 (29.0)	0.395
Clopidogrel	33 (26.6)	24 (25.8)	9 (29.0)	0.727
Warfarin	6 (4.8)	3 (3.2)	3 (9.7)	0.150
Cilostazol	7 (5.6)	5 (5.4)	2 (6.5)	0.824
NOAC	8 (6.5)	6 (6.5)	2 (6.5)	1.000
Statin	61 (49.2)	46 (49.5)	15 (48.4)	0.918
Laboratory data (median [range])				
Hemoglobin (g/dL)	9.6 [4.8–14.4]	9.5 [4.8–16.1]	9.9 [6.1–13.0]	0.158
Total lymphocyte count (10^3^/µL)	1160.0 [240–11,390]	1270.0 [240–11,390]	1000.0 [340–3220]	0.254
Platelet (000s)	189.0 [12–820]	191.0 [12–820]	167.0 [20–329]	0.221
CRP (mg/dL)	1.8 [0.1–40]	1.1 [0.1–40]	6.7 [0.1–40]	0.003
Total protein (g/dL)	6.1 [4.1–8.4]	6.1 [3.2–8.4]	5.9 [4.1–7.9]	0.383
Albumin (g/dL)	3.0 [1.6–4.4]	3.0 [1.5–4.4]	2.8 [1.6–3.8]	0.009
BUN (mg/dL)	54.3 [11.9–177.8]	53.0 [13.9–184]	54.9 [11.9–143.4]	0.944
Creatinine (mg/dL)	4.8 [0.7–31.4]	5.1 [0.7–32.9]	4.3 [0.9–11.7]	0.021
Total cholesterol (mg/dL)	127.0 [45–494]	126.0 [45–494]	135.0 [52–290]	0.835
Total calcium (mg/dL)	7.7 [5.0–10.5]	7.7 [5.0–10.4]	7.7 [5.3–10.5]	0.330
Phosphorus (mg/dL)	4.2 [1.5 –12.6]	4.1 [1.5–12.6]	4.2 [1.7–9.9]	0.606
Sodium (mEq/L)	136.5 [122–153.6]	136.6 [122–153]	136.4 [124–153.6]	0.471
Potassium (mEq/L)	4.3 [2.8–7.8]	4.3 [2.8–7.8]	4.3 [2.8–6.2]	0.976
BMI (kg/m^2^)	23.5 [15.1–41.9]	23.4 [15.1–41.9]	25.0 [16.0–39.2]	0.339
Body composition (median [range])				
ECW (L)	16.2 [8.4–32.5]	17.4 [8.4–32.5]	12.2 [10.3–30.7]	0.371
ICW (L)	15.3 [7.8–28.0]	15.8 [7.9–28.0]	12.5 [7.8–22.2]	0.000
TBW (L)	31.8 [16.3–56.4]	33.8 [16.3–56.4]	27.7 [19.7–52.9]	0.013
ECW/ICW ratio	1.13 [0.62–1.90]	1.05 [0.62–1.89]	1.22 [0.87–1.78]	0.001
LTI (kg/m^2^)	11.6 [5.3–21.4]	12.4 [5.8–21.4]	9.4 [5.3–16.4]	0.000
FTI (kg/m^2^)	10.7 [0.9–29.8]	9.7 [0.9–29.0]	11.2 [2.1–29.8]	0.042
LTM (kg)	28.2 [2.0–59.3]	30.7 [2.0–59.3]	22.9 [12.5–51.3]	0.013
ATM (kg)	24.8 [2.3–66.9]	24.0 [2.3–64.5]	29.5 [2.4–66.9]	0.222
FAT (kg)	18.4 [1.7–49.2]	17.6 [1.7–47.4]	21.7 [4.9–49.2]	0.042
Body cell mass	15.4 [2.3–36.1]	16.8 [2.3–36.1]	11.7 [4.1–29.6]	0.001
Dry weight (kg)	57.6 [33.5–99.7]	58.5 [33.5–99.7]	53.3 [37.2–90.9]	0.228
Information of initiating hemodialysis (median [range])				
Blood flow rate (mL/min)	200 [180–250]	200 [180–250]	220 [180–250]	0.370
Dialysis duration (h)	2.0 [1.0–4.0]	2.0 [1.0–4.0]	3.0 [2.0–4.0]	0.202
Ultrafiltration volume (L)	1.5 [0.2–4.0]	1.5 [0.3–4.0]	1.7 [0.2–4.0]	0.421

NOAC: non-vitamin K antagonist oral anticoagulant; CRP: C-reactive protein; BUN: blood urea nitrogen; BMI: body mass index; ECW: extracellular water; ICW: intracellular water; TBW: total body water; LTI: lean tissue index; FTI: fat tissue index; LTM: lean tissue mass; ATM: adipose tissue mass; FAT: fat mass.

**Table 2:** Multivariate Cox proportional hazard analysis of permanent catheter patency survival in the non-patent group.

	Hazard Ratio	95% CI of Difference		*p* Value
		Lower	Upper	
Model 1				
ECW/ICW ratio	6.016	1.895	19.094	0.002
Model 2				
ECW/ICW ratio	6.615	1.958	22.346	0.002
Age	1.049	1.016	1.083	0.004
Sex	0.950	0.458	1.971	0.890
Model 3				
ECW/ICW ratio	4.792	1.225	18.747	0.024
Age	1.043	1.006	1.080	0.021
Sex	1.211	0.566	2.592	0.622
LTI	0.907	0.768	1.071	0.248
FTI	1.031	0.962	1.104	0.393

HR = hazard ratio; CI: confidence interval; ECW: extracellular water; ICW: intracellular water; LTI: lean tissue index; FTI: fat tissue index.

## Data Availability

The data presented in this study are available on request from the corresponding author. The data are not publicly available due to privacy and ethical.

## Supplementary Materials

The following supporting information can be downloaded at: https://www.mdpi.com/article/10.3390/diagnostics13152545/s1. Figure S1: ROC analysis of E/I ratio, LTI and FTI for patients of patent and non-patent group. Figure S2: Kaplan-Meier survival curve between groups with high CONUT score (≥3) and low CONUS score (<2) Table S1a: Baseline characteristics of the study participants with infection subgroup. Table S1b: Baseline characteristics of the study participants with malfunction subgroup. Table S2: Univariate Cox proportional hazards analysis in non-patent group.
